# On the Nature of the Mother-Infant Tie and Its Interaction With Freudian Drives

**DOI:** 10.3389/fpsyg.2020.00317

**Published:** 2020-02-26

**Authors:** Michael Kirsch, Michael B. Buchholz

**Affiliations:** ^1^Institute of Physiological Chemistry, University Hospital Essen, Essen, Germany; ^2^Department of Social Psychology and Ph.D. Program, International Psychoanalytic University Berlin (IPU), Berlin, Germany

**Keywords:** affective bond, Bowlby, SEEKING, ghrelin, breast-feeding

## Abstract

The affective bond between an infant and its caregiver, the so-called mother-infant tie, was analyzed by various reputable psychologists (e.g., Ainsworth, Clark, Erikson, Anna Freud, Harlow, Klein, Spitz, and Winnicott) but both the basic tenets of the bond and the importance of the trauma of maternal deprivation for personality disorders in adults were introduced by Bowlby. Although Bowlby was a trained psychoanalyst, he rejected central cornerstones of Freudian theory (esp. drive theory) and used concepts promulgated by renowned ethologists (Tinbergen and Lorenz) to establish his framework of “instinctive behavior” that has been developed further into the concept of “attachment theory” under the influence of Mary Ainsworth. However, since any precise experimental facts were lacking when Bowlby formulated his ideas on the concept of instinctive behavior, the whole framework is a descriptive, category-driven approach (like the ones of Freudian drives). In order to connect the mother-infant tie – as propounded by Bowlby – with experimental data, this manuscript undertakes a biochemical analysis of it because this strategy proved somewhat successful in relation to Freudian drives. The analysis unfolded that the neurochemical oxytocin, released by the action of sensory nerves, is of utmost importance for the operation of the mother-infant tie. Furthermore, multiple evidences have been presented to the fact that there is strong interaction between unconsciously operating Freudian drives and the consciously acting mother-infant tie (that is now classified as a drive). The outlined interaction in conjunction with the classification of attachment urges as drives gave a very detailed insight into how a SEEKING-derived reward can be evoked during operation of the mother-infant tie. In summary, there is no need to marginalize either the mother-infant tie or Freudian drives but rather there is need to respect both (principally different) impulses in moving toward a more extensive description.

## Introduction

“*There is bad blood between psychoanalysis and attachment theory. As with many family feuds, it is hard to identify where the problem began*” (Fonagy, 2001, p. 1).

In 1951 Bowlby contended that the infant should experience a continuous relationship with its mother in order to ensure its mental health (Bowlby, 1951) and later he pointed out that a trauma of maternal deprivation (Bowlby, 1969, 1973, 1980, 1988) is responsible for many forms of psychoneurosis and character disorders. Of course, the importance of a loving relationship during the first years of life was mentioned by Sigmund Freud^1^. Since Harlow’s experiments studying the preference of new-born rhesus-monkeys (mother with…) scholars of various orientations studied infants behavior of human and prehumen neonates in order to understand more precisely the social dimension of the mother-infant tie. The Freudian hypotheses that a partial component of sexual drives, orality, determines the choice of an object, mother’s breast, driven by hunger, was to be differentiated: Harlow (1958) showed that rhesus-infants preferred not the surrogate mother delivering milk but the mother surrogate delivering a warm cuddly blanket. Thus, as infants prefer (temporarily) bonding to saturation, the nature of the mother-infant tie had to be thought anew. Influenced by such findings Bowlby was ambivalent to Freud’s life’s work (Bowlby et al., 1986, p. 45) and especially Freud’s “Trieblehre” (best translated as ‘theory of motivational drives’) was rejected by him because he believed that the ‘drive’ construct represented only a description of behavior (Bowlby, 1969, p.136).

In order to create an alternative theoretical framework to Freud’s theory of motivational drives, Bowlby had to answer the question about the nature of a disrupted mother-infant tie. Noticeably, by building on concepts from ethology^2^ and developmental psychology (Bretherton, 1992), Bowlby established that there is a strong mother-infant tie and that a disruption of it (so-called detachment)^3^ produces distress, anxiety and fear in the infant (Bowlby, 1958). Unbeknownst to Bowlby, simultaneously published rejoinders from Anna Freud (A. Freud, 1960), Max Schur (1960), and René Spitz (1960) torpedoed the theoretical framework of attachment theory which had been developed at that time. After this concerted protest Bowlby refrained from publishing his improved ideas in psychoanalytical journals, as is documented by two completed, unpublished manuscripts on defensive processes related to mourning (Bowlby, 1962a,b). In 1969 Bowlby classified the mother-infant tie as an instinct (as opposed to a drive) by replacing Freud’s definition of instincts (translated from the German noun “Instinkte”) as “*inherited mental formations*” (S. Freud, 1915b, p. 3017) with “*Instinctive behavior is not inherited; what is inherited is a potential to develop certain sorts of system, termed here behavioral systems,.”* (Bowlby, 1969, p. 45). Such a position is in line with Tinbergen’s instinct concept of Hierarchical Organization of Circuit Nodes^4^ (Tinbergen, 1950) which, of course, somewhat foreshadowed the development of Command Systems (Panksepp, 1998; Panksepp and Biven, 2012). However, by simply capturing the positions of the Nobel laureates Tinbergen and Lorenz in order to replace Freud’s framework, Bowlby would have faced some theoretical difficulties. At first, Tinbergen concluded “*The motivation of human behavior is a matter of much discussion. Here again, introspection is a hindrance to understanding: it reveals to us only conscious subjective phenomena, though we have learnt from Freud that non-conscious phenomena of a quite different nature are at work as well.”*(Tinbergen, 1989, p. 208)^5^. Thus, from the perspective of Tinbergen the concept of Hierarchical Organization of Circuit Nodes is a framework complementary to Freud’s theory of motivational drives because Tinbergen’s theory described conscious-dependent motivations whereas Freud established his framework in order to explain unconscious mental impulses. Secondly, Erich Fromm deployed some remarkable similarities between the theories of Freud and Lorenz on aggression (Fromm, 1972, 1973). Thus, Bowlby had founded his attachment theory on theoretical concepts of conscious motivations that were somewhat compatible with Freud’s unconscious drives. Because of this conclusion, Bowlby was under pressure either to develop an entirely new theory of motivational behavior or (what he obviously had done) to improve published concepts of renowned ethologists. The main rooted objection in Tinbergen’s framework was the intrinsic exclusion of any feedback stimuli that were believed to mainly mediate behavior (of animals) (Hinde, 1956). Bowlby obviously built -up his theory of instinctive behavior^6^ (of which attachment is a prime example (Boag, 2017, p. 47)^7^ by comprising the recommendations of Tinbergen (1950) and Lorenz and Leyhausen (1973) to incorporate external as well as internal stimuli for the description of (conscious-dependent) behavior. Bowlby respected the valid objection of missing feedback stimuli by introducing (at that time hypothetical) factors for attachment activation, attachment termination, and attachment inhibition (Bowlby, 1969, pp. 95–96)^8^. However, even an improved theory of conscious attachment behavior will run into difficulties by describing mental processes with an unconscious part.

Let us pass along some stations of the road to a social conception of human kind before we arrive at the main station of this contribution: to study the biochemical contributions to a “Bowlbyian drive.” We want to deliver a more precise understanding of attachment and bonding which might contribute to psychotherapeutic practice.

A shift was more and more realized: human beings are not “driven” by primitive needs like hunger, the search for intersubjectivity was more important for them right from the start. This shift was extended to *intersubjectivity before language* in the work of Meltzoff and his co-workers (Meltzoff and Moore, 1977, 1983; Meltzoff, 2002). They opened windows on preverbal sharing: New born infants observe another person’s movement of a body part (tongue protrusion) and they respond with the same body part movement. At some later age a *supramodal space* is created: infants respond with an equivalent but not the same body part; the authors speak of an “active intermodal mapping.” Greetings in adult life are often executed by one person saying “hallo” and the other responds with a tipping to the hat. The equivalent mode creates a common social space leaving enough freedom for the person’s choice how to respond. “Active intermodal mapping” was found in very early age. A next step is the observation that human babies do not only observe their mother’s face but they follow mother’s glance; a triadic space (mother – infant – observed object) is created which is considered an important forerunner of language use (Fivaz-Depeursinge et al., 2005, 2010). At the age of 9 months toddlers enter a stage which Tomasello (1999, 2003) named as “revolution.” Children understand that mothers are driven by intentions, observable is guided by internal processes and they fully understand the ideational motivation when they realize that they themselves are ideational agents. They point to objects while observing mother’s glance: does she follow the deictic imperative? The 9-month-revolution together with the triadic space based on “active intermodal mapping” realize what is termed “intersubjectivity” from which language acquisition arises (Liszkowski, 2006; Goldin-Meadow, 2011).

Meltzoff et al. (1999) could demonstrate that 18-months old children understand the plans of another person and that they do not follow observable behaviors: An experimenter cannot accomplish some tasks, such as pulling a string through wooden balls; if he leaves the utensils in front of the child, the infant does the task immediately. This cannot be explained by imitation learning; and further; if a technical machine tries the same as the experimenter the child loses interest after a few seconds. Children are bound to humans. One result shall be mentioned here: Infants who follow looks more often and perceive it, among other things, as a conscious-controlled process learn language better (Meltzoff and Brooks, 2007). The important role of gaze had been demonstrated very early by the groundbreaking research of Beatrice Beebe (2014). Before video-technique was at hand she observed by micro-analysis of a filmed mother-infant interaction that the mother for fractions of a second interrupted gaze exchange by gaze-stepping sideward. This motherly behavior (called “chase-and-dodge”-pattern) could predict from the age of 4 months an attachment disorder in the child at the age of one year. Later she found that patterns of vocalization rhythms were as important as such patterns of gaze for mature face-to-face conversations in later age (Jaffe et al., 2001).

This bonding could be profoundly demonstrated in the so-called still-face experiments by Tronick (2007). A mother and a 4–6 month old baby in a seat are videotaped playing and giggling together. After a while the mother follows the instruction to turn her head away and when she returns to look unmoved onto a fix point besides the baby. She is instructed for 2 min not to respond to the baby’s attempts to attract her attention. From the series of observations Tronick made only one is to mention here: The baby points to certain objects in the common environment with the mother, although pointing gestures are observed under normal circumstances only some months later.

After these two phases of the experiment the mother returns to her normal playful interaction with the baby. Ham and Tronick (2009) measured the physiological responses of mother and baby in the three phases of the experiment. The most important result is that the mother in the “still” phase of the experiment has a very high level of arousal which she tries to control by her breathing. When returning to the 3rd phase she adapts her breathing rhythm to that of the child and makes attempts to demonstrate the child to calm down by controlling common breathing. This reestablishment of the common tie is realized by controlled rhythmical breathing which cannot be realized without some biochemical responses. Here is (for this manuscript) an important extract of Tronick’s theory:

“*Infants must collaborate with others to successfully regulate their physiological homeostatic states. Of course, the infant is a bounded organism and obviously the adult is external to the infant’s (anatomical) boundaries. Nevertheless, the adult is part of the infant’s homeostatic regulatory system, as much a part as any internal regulatory process*” (Tronick, 2007, p. 403 f.)

Even temperature is regulated by a “dyadically expanded state of consciousness,” just because the infant cannot accomplish this by himself and Tronick generalizes: “*Thus the infant’s physiological state is always in some part dyadically regulated with the caregiver, an external component of the infant’s regulatory system*” (p. 404). It is communication that guides this dyadic emotional regulatory system, communication via gaze, small sounds of consolation or reassurance, touching the baby’s head etc. Mother’s activities are responded to by the baby’s responses and are evaluated by her as helpful and calming – or not, in which case she alters her activities. Both create a mutually regulated process, where miscommunications are normal, if taken as pertinent for change and learning. Such a “dyadic state of consciousness,” as Tronick terms it, creates a paradox: “*On the one hand, more information is integrated into the system, making it more complex, while on the other hand, the increase in coherence results in a more organized state.”* (p. 406). In humans this process is just as importantly dyadic. It is a process involving two minds. Two seemingly contradictory goals are achieved: “*When infant and mother mutually create this dyadic state – when they become components of a dyadic system – both fulfill the first principle of systems theory of gaining greater complexity and coherence*.” (p. 407).

Here, system theory equips us with new ways of thinking the bonding resp. attachment between mother and child. Temperature regulation, gestures, gazes and many other bodily exchanged components can be viewed as elements of a dyadic state creating mutual awareness and consciousness forming the base of interaction and intersubjectivity at a very early level. This has been thought as attachment or bonding by John Bowlby. In what follows we want to contribute some biochemical observations completing the interactive sociality of a body-mind process. What’s going on in the body complements, what can be observed in interaction between Freudian drives and attachment urges, and what Command Systems are involved. How relevant these considerations are can be concluded when considering the work of Feldman (2012) and Jobst (Jobst et al., 2015): in later age social exclusion can be demonstrated to have effects on temperature regulation, attentiveness to new tasks and emotion regulation (depressed self-esteem).

## General Restrictions

The evaluation of the mother-infant tie is a topic of many scientific disciplines and because of this fact a number of parameters have been identified with key importance for the mother-infant tie in general, e.g., adrenergic systems, endorphin signaling, and the importance of the human father. However, here we are interested on possible interactions between Freudian drives and the mother-infant tie. The processing of both Freudian drives and the mother-infant tie can be described as a cascade of biochemical metabolites. In order not to be confused by an armada of metabolites such a cascade is viewed as a biochemical stream. The first metabolite is the “spring” of such a biochemical stream. All other metabolites of such a cascade can be classified as down-streaming products. In this manuscript it is suggested that the interaction between Freudian drives and the mother infant tie can proceed effectively at the “springs” or at very early down-streaming products. Adrenalin and especially endorphins are late down-streaming products of both Freudian drives and the mother-infant tie. Such late down-streaming metabolites are often responsible for achieving (essential) feed-back. Since the focus of this manuscript is the interaction between Freudian drives and mother-infant tie and also due to limited space, we must restrict the detailed biochemical evaluation to very early down-stream metabolites. Astute readers should further note that other social bonds (e.g., pair bonding) are - at the level of neuroanatomy, (neuro)endocrinology and biochemistry - quite different from the mother-infant tie. Thus, the mother-infant tie cannot be viewed as a characteristic example of attachment.

## Up-Date of Freud’s Theory of Motivational Drives

A comparison between Bowlby’s mother-infant tie and Freudian drives can only be done with the up-dated version of the latter (Kirsch and Mertens, 2018; Kirsch, 2019) which is now briefly expressed: In 1905 Freud descripted precisely the onset of his drives: “*The source of an instinct is a process of excitation occurring in an organ and*⋯” (S. Freud, 1905, p. 1492). Thus, a Freudian drive had to be initiated by an excited organ that must not be the brain. In the case of the sexual drive Freud excluded explicitly the brain as the drive source: “*It seems probable, then, that special chemical substances are produce in the interstitial portion of the sex-glands; these are then taken up in the blood stream and cause particular parts of the central nervous system to be charged with sexual tension*.” (S. Freud, 1905, p. 1530). Conclusively, Freud described here that the onset of the sexual drive is initiated by an organ-dependent chemical messenger that addresses the brain. Freud was probably unable to use the term “hormone” which would have been appropriate for this proclamation because this term was coined in the same year by Starling: “*These chemical messengers, however, or “hormones” (.), as we might call them, have to be carried from the organ where they are produced to the organ which they affect by means of the blood stream and the continually recurring physiological needs of the organism must determine their repeated production and circulation through the body*.”(Starling, 1905, p. 340). Thus, the chemical messenger mentioned by Freud can be classified as a peripheral hormone. Noticeably, the hypothesis of such hormone-controlled human drives^9^ was in conflict with the hypothesis of decisive brain-dependent (metabolic) sensing mechanisms, as has been firstly mentioned by Bernard for the onset of the hunger drive (Bernard, 1849). In 1915 Freud offered the architecture of his drives: “*We are now in a position to discuss certain terms which are used in reference to the concept of an instinct – for example, its* “*pressure*,” *its “áim,” its “object” and its “source.” By the pressure [Drang] of an instinct we understand its motor factor*,…*”* (S. Freud, 1915a, p. 2960). Inspired by Freud’s view to respect hormones as principle driving force of his drives (and by correcting some mistranslations^10^), it was possible to link imperative motor factors with known hormones^11^ (Table 1, *vide infra*) (Kirsch and Mertens, 2018). The view that hormones are responsible for the onset of a drive is not in conflict with Freud’s claim that the “*aim [Ziel] of an instinct is in every instance satisfaction*” (S. Freud, 1915a, p. 2960). During satisfaction all Freudian drives secreted the brain chemical 5-hydroxytryptamine (Kirsch and Mertens, 2018). This intermediate exerts a stimulatory control over pituitary release of β-endorphin in human beings (Petraglia et al., 1987; Maes et al., 1996) and endorphins are known to induce euphoria and to limit pain (Roth-Deri et al., 2008; Charbogne et al., 2014; Veening and Barendregt, 2015). The imperative motor factors address (not only one but) a variety of brain areas that were important for a specific drive activity (Kirsch, 2019). In correspondence with Freud’s perspective, there is no need to permanently monitor metabolic deficits with brain-dependent sensing activities for the onset of a drive.

**TABLE 1:** Biochemical interaction between Bowlby’s mother-infant tie and Freudian drives.

**Parameter**	**Bowlby’s mother-infant tie**	**Freudian drives**
Initiating activation factor	oxytocin (women and infants) vasopressin? (men)^a^	adenosine (sleep)^b^ angiotensin II (thirst) ghrelin (hunger) testosterone (sexual drive)
Source	excitation of sensory nerves	excitation of organs
Intensification	(self-intensification) oxytocin (intensification by amines) 5-hydroxytryptamine^c^ (intensification by imperative motor factors) angiotensin II at birth ghrelin at postnatal periods	
Inhibition factor	testosterone (mother only)	competing Freudian drive^d^
Termination factor	adenosine (sleep) β-endorphin	5-hydroxytryptamine oxytocin (hunger)
Drive specific area	*caudal nucleus tractus solitaries*	*arcuate nucleus* (hunger)^b^ *subfornical organ, area postrema* and *organosum vasculosum of lamina terminalis* (thirst) *medial preoptic area* (sexual drive) *tuberomammillary nucleus* (sleep)

*^*a*^, Feldman and Eidelman found a causal role of the initial mother–infant tie immediately after birth for the effectiveness of the father-infant bond which was typically developed during the third month of infant’s life (Feldman and Eidelman, 2007). The authors cannot find any reliable data sets with a man as exclusively caregiver after birth. Therefore the impact of oxytocin in a father-infant tie is unknown. ^*b*^, Reference: (Kirsch and Mertens, 2018). ^*c*^, Dopamine and noradrenaline can also intensify oxytocin signaling (*vide infra*). ^*d*^, Reference: (Kirsch, 2019).*

## Affective Neuroscience

The neuropsychologist Jaak Panksepp hypothesized (i.e., the so-called “opioid hypothesis”) that simultaneous releases of brain opioids in two interacting individuals are responsible for the formation of social attachment and corresponding emotions (Panksepp et al., 1978, 1980). In Panksepp (1992) introduced four biological brain based action system (expectancy, fear, rage and panic) and in 1998 he (Panksepp, 1998) assumes that our consciousness does come in subcortical regions of the brain, in innate, basal emotional systems. Panksepp (Panksepp, 1998, 2018; Panksepp and Biven, 2012; Solms and Panksepp, 2012) classifies seven different types of motivations that can evoke special behaviors, e.g., *seeking* for rewards/resources/sexual partners, *lust*, *caring* and affection, loss and *panic*, *rage*, *fear* and *play*. Special subcortical regions of the brain are involved with the processing to the corresponding emotions, which are classified as so-called Command Systems (labeled SEEKING, RAGE, FEAR, LUST, CARE, PANIC and PLAY)^12^. The SEEKING system is of particular significance for the up-dated theory of Freudian motivational drives in this context because it refers to general positive motivations (Wright and Panksepp, 2012). The satisfaction of a Freudian drive can release via intermediary 5-hydroxytryptamine endogenous opioids (such as β-endorphin) which are known to evoke euphoric mental states (*vide supra*).

Panksepp and Watt noted that the organization of emotions is much more complex than only addressing typical brain areas of Command Systems. They stated that “*the pressures of evolution*” stamped phylogenetic “*imprints*”, i.e., layered anatomical structures (so-called nested hierarchies) that controlled brain functionality via top-down interactions and bottom-up ones of two neighboring layers (Panksepp and Watt, 2011, p. 387) introduced a nomenclature for the functioning of these imprints that explains emotions on primary-process (arise from activation of subcortical areas), on secondary process (arise from Pavlovian and instrumental learning principles), and tertiary process brain activities (arise from neocortical interactions with paralimbic and limbic structures).

The authors are somewhat ambivalent to this concept. On the one hand, all imperative motor factors have targets in key regions of the Command Systems (esp. in brain areas of the SEEKING System) and can modulate all Command Systems in either a direct or an indirect manner (Kirsch, 2019). Because of such an addressing and the possibility of bottom-up interactions, the framework of Affective Neuroscience seems to explain conclusively the neurophysiological effects of Freudian drives to induce even tertiary process brain activities. On the other hand, authorities of Affective Neuroscience stated that Command Systems are activated by mechanisms that are incompatible with Freud’s perspective. Panksepp had been followed the view that homeostatic drives, in principle a metabolic deficit that is predicted to operate as a regulatory imbalance, can serve as inputs for the SEEKING system (Panksepp, 1998; Panksepp and Biven, 2012; Wright and Panksepp, 2012). The term homeostasis describes the regulation of an internal state (Berridge, 2004) and was introduced in 1925 by Cannon (1932) who picked –up the suggestion of Bernard (1849) (*vide supra*). Noticeably, homeostatic drive constructs were mainly used by behavioral oriented scientists (Berridge, 2004) but not respected by Freud (*vide supra*). The homeostasis construct, in the case of Affective Neuroscience the so-called need-detector mechanism (Panksepp, 1998; Solms, 2019), requires one (or more) central mechanism(s) in order to detect and counteract the metabolic deficit (Berridge, 2004). In Mayer’s (1958) glucostatic hypothesis postulated that plasma glucose levels are sensed by glucose receptors in the (*lateral*) *hypothalamus*. Solms and Turnbull seized Mayer’s suggestion for the need-detector mechanism construct: “*There is a range of need-detector mechanisms in the hypothalamus*… *They are not entirely specific, but the important issue is that these hypothalamic systems generate “needs”, and these “needs” activate the SEEKING system*… *Different hypothalamic regions switch these detector systems on (they act like “accelerators”) and off (acting like “brakes”)*…*Conversely, lesions to the “accelerator” system create an almost total loss of interest in food. Anorexia follows rapidly, although the animal will occasionally nibble-just enough to remain alive.*” (Solms and Turnbull, 2002, ch. 4). Thus, it is predicted that the first signal for the onset of a drive is generated in a special area of the brain in contrast to Freud’s perspective of launching motivational drives (*vide supra*). Because of this incompatibility of the need-detector mechanism construct, we need to use hormone-controlled drives for inputs of the Command Systems in order to be compatible with Freud’s perspective.

## (Un)Conscious Drives

For Freud drives “*represent an instigation to mental activity*” (S. Freud, 1926, p.4343; Holder, 1970, pp. 19) and a drive-dependent activation of the SEEKING System is fully in keeping with Freud’s view. Since the activation of the SEEKING system is accompanied with the release of the neurochemical dopamine (Panksepp and Biven, 2012; Wright and Panksepp, 2012), we need to suggest that the release of dopamine in the *nucleus accumbens* should represent a characteristic read-out parameter of a drive. Freud clarified that his drives operate in an unconscious manner: “*I am in fact of the opinion that the antithesis of conscious and unconscious is not applicable to instincts. An instinct can never become an object of consciousness* – *only the idea that represents the instinct can.*” (S. Freud, 1915b, p. 3000). Bowlby noted that the intensity of the mother-infant tie can be dependent on an infant-mediated visual monitoring of his mother (*vide supra*) and such a process require awareness via the action of sensory nerves. Therefore, by evaluating possible interactions between Freudian drives and the mother-infant tie, the authors followed Tinbergen’s view that the onset of a drive can be achieved in an unconscious manner (Freudian drive type) or in a conscious one (attachment type). (Adolphs and Anderson, 2018, p. 298) noted, that the term ‘unconscious’ is frequently used for unawareness of an external stimulus at subliminal levels (e.g., Shervin and Fritzler, 1968; Greenwald et al., 1996). This interpretation of unconsciousness (unawareness of an external stimulus) seems to be very common in neuroscientific disciplines, e.g., “*In one of Nobel-Prize-winning neuroscientist Roger Sperry’s famous cases, pornographic pictures were projected to the isolated right hemisphere of a patient. The patient blushed and giggled. When Sperry asked her why she was embarrassed, she was unable to account for it. This case (..) demonstrates that an entire cerebral hemisphere can process information “unconsciously.*” (Solms and Turnbull, 2002, ch. 3). In contrast, by using an operating Freudian drive as a typical example of unconsciousness, it can be assumed that an unconscious action requires an internal stimulus^13^ –, and not an external one –, in order to initiate a mental process in a subconscious manner (*vide supra*). Panksepp and Biven (2012, p. 8) noted that consciousness require the intermediation of neuromodulator/neurotransmitter codes and the brain chemical dopamine can be classified as such a compound (e.g.,Webster and Stanford, 2001). The unconsciously operating Freudian drives require the intermediation of (peripheral) hormone codes (i.e., imperative motor factors) for their onsets (Kirsch, 2019). Peripheral hormones are not, according to the notion of Panksepp and Biven (2012, p. 8), directly involved in the onset of consciousness. A Freudian drive can only reached (pre)conscious levels when the corresponding imperative motor factor can occupy its targets (hormone receptors) on various pre-synapses thereby inducing the release of neurotransmitters or neuromodulators (such as dopamine) (Kirsch, 2019). Conclusively, all types of drives are predicted to activate SEEKING via dopamine release in the *nucleus accumbens*. Unconscious Freudian drives must achieve this release via an internal stimulus, i.e., a stimulus (such as a peripheral hormone) that is not directly required for consciousness whereas a conscious drive (like the suggested mother-infant tie) must activate sensory nerves in order to stimulate *accumbal* dopamine release.

## Oxytocin as Initiating Chemical Messenger of Attachment

Bowlby’s proclamation of a factor for attachment activation (as well as the suggestions of factors for attachment termination and attachment inhibition) intrinsically predicted that an initiating chemical messenger^14^ facilitates the onset of attachment behavior as did imperative motor factors for the onset of Freudian drives. There is general consensus that the intermediacy of the neuropeptide oxytocin – that is produced in the *paraventricular*, *supraoptic*, and *accessory nuclei* of the *hypothalamus* (Insel, 1992) – is an important factor in regulating (adult) social behaviors of all (female)^15^ vertebrates (McCarthy, 1990; Insel and Young, 2001) including human beings (Carter, 2018; Ebert and Brüne, 2018; Kendrick et al., 2018; Quintana et al., 2019). Bowlby’s postulation – that the strong mother-infant tie can be directed by external stimuli (*vide supra*) – presumed that the initiating chemical messenger of attachment is simultaneously released in both individuals in order to evoke isochronally positive emotions (Feldman and Eidelman, 2007) that build -up the affective bond. Such a concurrent release of oxytocin would require the presence and simultaneous activation of sensory nerves in each individual. In fact, different kinds of sensory nerves in the mother’s breast, the infant’s oral mucosa and the skin of both individuals connect to the *nucleus tractus solitaries* and the *caudal nucleus tractus solitaries* can activate oxytocin-producing neurons in the *supraoptic nucleus* of the *hypothalamus* (Raby and Renaud, 1989)^16^. Accordingly, studies conducted on experimental animals demonstrated that the release of oxytocin can be the decisive factor for establishing the mother-infant tie^17^. In addition, an impaired oxytocin signaling affected attachment behavior of human infants (Chen et al., 2011). Although the oxytocin release kinetic evoked by skin to skin contact is quite different from the one induced by breast-feeding (Uvnäs Moberg and Prime, 2013), it is nevertheless concluded that oxytocin is the initiating chemical messenger of the mother-infant tie (Table 1).

A surprising finding transpired when evaluating the importance of internal stimuli for central oxytocin release. Noticeably, the amount of oxytocin liberated by the action of sensory nerves is most likely intensified for establishing the mother-infant tie^18^ probably via two mechanisms. Firstly, a self-intensifying mechanism^19^ because oxytocinergic neurons expressed targets (so-called receptors) for oxytocin (Yoshimura et al., 1993; Freund-Mercier et al., 1994) and occupation of these receptors facilitates the firing of oxytocinergic neurons (Yamashita et al., 1987; Ingram et al., 1998).

**FIGURE 1:**
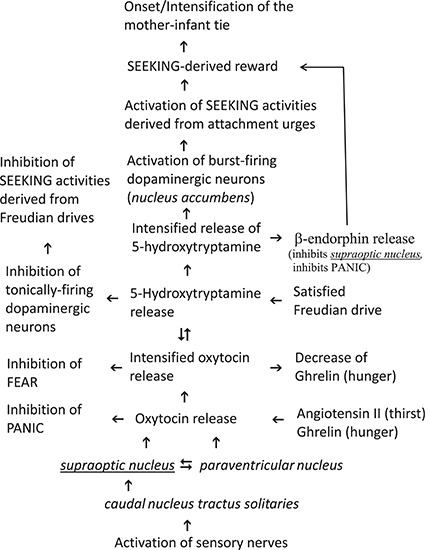
Proposed onset of the Mother-Infant tie from the perspective of the infant.

The second potential oxytocin intensifying mechanism requires an intermediate. It is known that high amounts of exogenously administered oxytocin leads to the central release of noradrenaline, dopamine and (especially of) 5-hydroxytryptamine^20^, respectively (Pfister and Muir, 1989; Onaka et al., 2003). Interestingly, these central amines can activate oxytocinergic neurons (Badgy and Kalogeras, 1993; Onaka et al., 2003; Love, 2014). In summary, there are two intensifying circuits for oxytocin signaling: the self-intensifying one (i.e., oxytocin release leads directly to additional oxytocin release) and the alternative mechanism with intermediary operating amines^21^ (i.e., oxytocin release leads to amine release thereby evoking the release of additional oxytocin). The latter alternative mechanism of oxytocin intensifying offers the somewhat surprising possibility that a satisfied Freudian drive can principally support attachment urges by enhancing the concentration of 5-hydroxytryptamine (Table 1). Noticeably, a satisfied Freudian drive is characterized by the release of 5-hydroxytryptamine (Kirsch and Mertens, 2018; Kirsch, 2019). This intermediate should be involved in the establishment of the mother-infant tie because there are strong indications that a disturbed signaling of 5-hydroxytryptamine in the forebrain during the early postnatal period can lead to increased anxiety-like phenomenon in the adult (Gross et al., 2002). In addition, the importance of 5-hydroxytryptamine in the development of affective bonds cannot be marginalized because a defect in a special 5-hydroxytryptamine receptor gene is connected with the psychological disorder referred to as ‘avoidant attachment’ (Gillath et al., 2008). There is further evidence that coordinated activity between oxytocin and 5-hydroxytryptamine is required for the [SEEKING-dependent (Figure 1, *vide infra*)] reward associated with social interactions (Dölen et al., 2013). Thus, a satisfied Freudian drive can (but does not necessarily have to) intensify attachment behavior by increasing the concentration of intermediary 5-hydroxytryptamine. Conclusively, noradrenaline, dopamine and especially 5-hydroxytryptamine can be classified as intensifiers of oxytocin release (Table 1).

## The Interaction Between Oxytocin and Imperative Motor Factors

Of course, a variety of signal molecules modulate oxytocin secretion [for instance caffeine results in the release of oxytocin (Wu et al., 2017)], but in this manuscript the search for such chemical messengers must be restricted to imperative motor factors. Although hypothalamic biosynthesis of oxytocin depends on the intermediacy of estrogens like estradiol (i.e., a down-streaming product of testosterone), most of the oxytocinergic neurons do not express a receptor for this steroid hormone on their surfaces (Jirikowski et al., 2018) and because of this missing target an efficient release of oxytocin induced by estradiol is unlikely^22^. Thus, estradiol is involved in the biosynthesis of oxytocin and in the optimization of oxytocin receptors but estradiol is of minor importance for the oxytocin release.

Behavioral studies demonstrated that testosterone reduced the attention of human mothers to their infants and that this temporary ignoring can be overcompensated by the administration of oxytocin (Holtfrerich et al., 2016, 2018). These findings are in line with the Steriod/Peptide theory of social bonds (van Anders et al., 2011). This theory predicted that high amounts of oxytocin in combination with high amounts of testosterone facilitate sexual interest by decreasing maternal care (Gordon et al., 2017). Thus, testosterone inhibits maternal behavior in a reversible manner and may therefore be classified tentatively as an attachment inhibition factor (Table 1). Noticeably, as testosterone concentrations in human infants are very low (only around 3 nM (Kai et al., 2007), newborn infants are most likely unable to inhibit the mother-infant tie via the intermediacy of this steroid hormone.

The imperative motor factor of sleep (i.e., adenosine) strongly inhibits oxytocin release (Wang et al., 2002) and can be classified as an attachment termination factor (Table 1)^23^.

The interaction between ghrelin (i.e., the imperative motor factor of hunger) and oxytocin is at present a topic of (neuro)endocrinological research. Firstly, the administration of ghrelin in experimental animals increased the oxytocin concentration in line with the dose given (Szabo et al., 2019), and ghrelin can therefore be classified as an intensifier of oxytocin release. Noticeably, enhanced ghrelin levels are present in human infants and in their mothers’ breast milk (Savino et al., 2011; Shilina et al., 2011; Kon et al., 2014). Secondly, oxytocin decreases ghrelin levels in healthy males (Vila et al., 2009), promote termination of feeding in untrained animals (Arletti et al., 1989; Herisson et al., 2016; Head et al., 2019) and can thus be classified as a hunger termination signal (Table 1)^24^. However, the imperative motor factor of thirst, i.e., angiotensin II, can be also classified as an attachment intensifying molecule. Angiotensin II stimulates the release of oxytocin (Lang et al., 1981; Geddes et al., 1994) and plasma in a human newborn has high concentrations of angiotensin II (Pipkin and Symonds, 1977). Thus, the intermediacy of the imperative motor factor of thirst at birth in the neonate (and probably also the one of hunger during the postnatal period) seems to be important for “early” release of oxytocin^25^ at high levels in order to establish a strong mother-infant tie (Table 1)^26^.

In summary, imperative motor factors can directly and indirectly intensify oxytocin release in the neonate. The imperative motor factors of thirst and hunger can directly intensify oxytocin release, all imperative motor factors can indirectly intensify oxytocin release via the intermediacy of 5-hydroxytryptamine, that is always secreted during Freudian drive satisfaction. The ability of imperative motor factors of hunger and thirst to directly increase oxytocin secretion is putatively important for the infant both at birth and during the postnatal periods because the concentration of angiotensin II is high at birth and the concentration of ghrelin is high in human milk during the first 2 weeks of the postnatal period. In contrast, the imperative motor factor of sleep decreases oxytocin secretion and can be classified as an attachment termination factor whereas testosterone (sexual drive) inhibits maternal behavior. Noticeably, the inhibitory action of testosterone on the mother-infant tie cannot proceed in the infant.

## The Action of Oxytocin on Command Systems

Oxytocinergic neurons originating in the *paraventricular nuclei* of the *hypothalamus* send projections to the *ventromedial nucleus* (RAGE and LUST) (Tribollet et al., 1992; Sabatier et al., 2007), to the *arcuate nucleus* (drive specific area of hunger) (Maejima et al., 2014), to the *ventral tegmental area* (SEEKING) (Shahrokh et al., 2010), to the *nucleus accumbens* (SEEKING) (Ross et al., 2009), and to the *central amygdala* (FEAR) (Huber et al., 2005). Oxytocinergic neurons have also been identified in the *medial amygdala* (RAGE), in the *basolateral amygdala* (FEAR), and in the *bed nucleus of the stria terminalis* (RAGE, LUST, CARE, PANIC) (Mitre et al., 2018). In order to delineate between differences in oxytocin signaling between adults and infants, the distribution of oxytocin receptors (in brains of experimental animals) has been carefully evaluated. The oxytocin receptor distribution in the brain of adult rats is, as expected, nearly identical to the distribution of projections of oxytocinergic neurons, i.e., projections of oxytocinergic neurons and regions of oxytocin receptors are found in identical brain areas (Grinevich et al., 2015; Mitre et al., 2018). Interestingly, a transient expression of oxytocin receptor distribution was evaluated in brains of infant rats and the occurrence of oxytocin receptors in the *anterior thalamus* (PANIC) was only evident during early postnatal life (Grinevich et al., 2015). Panksepp and Biven noted that the PANIC command system can be downregulated by endogenous opioids, prolactin, and oxytocin (Panksepp and Biven, 2012, p. 325). Thus, the transient expression of oxytocin receptors in the *anterior thalamus* strongly indicated that a down-regulation of the PANIC command system via oxytocin can be effective only in the brains of (rat) infants.

In order to classify which of the aforementioned brain areas are key to the mother-infant tie (beside the *anterior thalamus* in infants, *vide supra*) in human beings, observations evaluated using functional magnetic resonance imaging should be most informative. In fact, mothers seeing photographs of their own children used the *amygdala* (FEAR and/or RAGE) and the *ventral tegmental area* (SEEKING) beside other brain regions that are not members of the Command Systems (Bartels and Zeki, 2004; Leibenluft et al., 2004; Strathearn et al., 2009; Wittfoth-Schardt et al., 2012). Functional magnetic resonance imaging studies have also demonstrated that exogenously administrated oxytocin results in deactivation of the *amygdala* when human mothers hear their infants laughing (Riem et al., 2012)^27^. Since there is evidence that oxytocin decreases the activity of the *central amygdala* (Huber et al., 2005; Viviani et al., 2011), it can be concluded that oxytocin-dependent modulation of the Command Systems PANIC, FEAR, and SEEKING are important for the build –up of the mother-infant tie in the infant and FEAR and SEEKING for the construction of the tie in the mother. The inclusion of the Command System CARE for the build –up of the mother-infant tie in the mother cannot be verified by experiments because CARE shares (as documented in footnote 12) all its important brain areas with other Command Systems (*vide supra*).

## The Importance of the Down-Streaming Product β-Endorphin

Since a satisfaction of a Freudian drive can lead to well-being or even euphoria, endorphins, which are known to induce euphoria and to mediate pain control (Roth-Deri et al., 2008; Charbogne et al., 2014; Veening and Barendregt, 2015), are connected with the drives of interest. Remarkably, this connection can also be constructed from the perspective of endorphins, because Henry noted in 1982: “*when the endorphin system is hypoactive*,……, *an increased drive ensues to satisfy a deprived state, whether this is an appetite for food, water, social contact, or sexual satisfaction, etc.* “(Henry, 1982, p. 239). β-Endorphinergic neurons are mainly located in the hypothalamic *arcuate nucleus* (Zangen et al., 2002) and the μ-opioid receptor is a typical target during β-endorphin neurotransmission (Bodnar and Klein, 2005). According to Panksepp all brain chemicals able to activate the μ-opioid receptor were “*incredibly effective*” in down-regulating PANIC (Panksepp and Biven, 2012, p. 325)^28^. There is evidence that well-being derived from social contacts require coordinated activity of *nucleus accumbens* oxytocin and 5-hydroxytryptamine (Dölen et al., 2013). The latter intermediate exerts a stimulatory control over pituitary release of β-endorphin in human beings (Petraglia et al., 1987; Maes et al., 1996) via its target 5-HT_1A_ (Navines et al., 2008). This receptor is occupied by donating exogenous oxytocin (Mottolese et al., 2014). Conclusively, β-endorphin is released during both satisfaction of a Freudian drive and processing of the mother-infant tie via 5-hydroxytryptamine dependent occupation of the receptor 5-HT_1A_ located on β-endorphinergic neurons. In other words, β-endorphin is a down-streaming product of both Freudian drives and the mother-infant tie. Although the complex cross talk between endorphins and oxytocin needs to be further evaluated, there are some indications that inhibitory μ-opioid receptors are present in the *supraoptic nucleus (but not in the paraventricular nucleus*) (Kovatsi and Nikolaou, 2019). The decreased oxytocin release can be evoked presynaptically by inhibition of noradrenergic endings in the *supraoptoic nucleus* but not in the *nucleus tractus solitaries* (i.e., the suggested drive specific area of the mother-infant tie) (Leng et al., 1995). An anonym reviewer mentioned that noradrenergic communications should be important when detachment result in post-traumatic situations (de Berardis et al., 2015). Thus, the oxytocin-dependent release of β-endorphin may be classified as a termination (or alternatively as a negative feed-back) signal for oxytocin secretion. Various other functions of endorphins in psychological situations cannot be covered here but can be found from works of Berridge (e.g., Smith and Berridge, 2007; Berridge, 2009; Berridge and Kringelbach, 2015; Castro et al., 2015).

## The Importance of the Seeking Command System for the Mother-Infant Tie

As “*the SEEKING system has been most closely associated with dopamine release*” (Wright and Panksepp, 2012, p. 11), any disturbance in central dopamine signaling would impair attachment urges when the SEEKING command system is of key importance. In fact, a polymorphism of one central target of dopamine (i.e., the dopamine receptor D2) has been linked to so-called ‘anxious attachment’ (Gillath et al., 2008). By accepting the view that oxytocin is the initiating attachment activation factor for the mother-infant tie, there are in principle two possibilities as to how oxytocin is able to increase central dopamine release. The first possibility would require targets for oxytocin on dopaminergic neurons that release dopamine after occupation of such targets. Unfortunately, this possibility has not been very well investigated, and any conclusive details are not in evidence. The second possibility would require the intermediacy of an oxytocin-dependent intermediate that can evoke dopamine release. Since the intermediacy of 5-hydroxytryptamine is also important for attachment urges (*vide supra*), it looks likely that this brain chemical mediates the release of dopamine for the onset of attachment (Figure 1, *vide supra*). This plausible rationalization seems to be in conflict with the fact that Freudian drives increases central 5-hydroxytryptamine levels in order to down-regulate dopamine (and thereby the activity of the SEEKING command system). Dopaminergic neurons can be classified into three categories (Goto et al., 2007): (A) inactive neurons; (B) tonically-firing neurons; (C) burst-firing neurons. Whereas a tonic activity reflects general states of motivation (Boureau and Dayan, 2011) (as should be the case with operating Freudian drives), the precise sense of phasic firing neurons is not fully understood although various explanations have been presented (Floresco et al., 2003; Goto et al., 2007; de Deurwaerdere et al., 2018). The whole interpretation is now complicated by the fact that there are at least fourteen different receptor types for 5-hydroxytryptamine (Boureau and Dayan, 2011). In order to prevent getting into the labyrinth of possibilities, the view is limited to the well evaluated 5-hydroxytryptamine receptors 5-HT_2A_ and 5-HT_2C_, respectively. Both receptors exert opposite control of dopamine release in the brain areas of the SEEKING system (Di Matteo et al., 2008, 2009) and both were constitutively (i.e., permanently) active (Berg et al., 2005). Whereas an occupation of the receptor 5-HT_2C_ leads to a tonical inhibition of dopamine release, an occupation of the receptor 5-HT_2A_ is associated with increased impulsivity (Boureau and Dayan, 2011). It can therefore be concluded that the receptor 5-HT_2C_ is expressed on tonically-firing dopaminergic neurons whereas the receptor 5-HT_2A_ is present on burst-firing dopaminergic neurons. At this state of knowledge the highly unlikely possibility cannot be excluded that the activation of phasic-firing dopaminergic neurons would disturb the inhibition of tonically-firing ones. Fortunately, the affinities of both receptors to 5-hydroxytryptamine have been evaluated with K_m_ values of 5.7 nM for 5-HT_2C_ and of 16 nM for 5-HT_2A_ (de Deurwaerdere et al., 2018). Thus, the tonically-firing dopaminergic neurons are half-maximally inhibited at a concentration (of 5.7 nM) where an occupation of the receptor 5-HT_2A_ on phasic-firing dopaminergic neurons can maximally achieve an efficiency of about 18%. Because of this rationalization the activation of phasic-firing dopaminergic neurons does not disturb the inhibition of tonically-firing neurons although only one intermediate (i.e., 5-hydroxytryptamine) operates at identical local areas of the SEEKING system. Thus, the inhibition of tonically-firing dopaminergic neurons and the activation of phasic-firing ones mediated by 5-hydroxytryptamine can be classified as a complementary action.

Conclusively, it can be now stated that Freudian drives increases 5-hydroxytryptamine to relatively low levels in order to down-regulate tonically-firing dopaminergic neurons whereas mother-infant tie dependent urges increases 5-hydroxytryptamine to relatively high levels in order to up-regulate burst-firing dopaminergic neurons. Whereas the former mechanism would deactivate SEEKING activities of Freudian drives, the latter would activate this Command System for urges of the mother-infant tie (Figure 1, *vide supra*).

Since Bowlby always vigorously disputed the usefulness of the drive construct (*vide supra*), he alternatively postulated attachment as a special kind of instinctive behavior (e.g., Bowlby, 1982, p. 39). However, by claiming attachment to be a drive the problem of drive-specificity needs to be addressed^29^. In order to sustain drive-specificity, Freudian drives activate neurons of both a drive-specific brain area and a brain area of the SEEKING system (i.e., an area that did release dopamine) (Kirsch and Mertens, 2018). In contrast to Freudian drives, the initiating attachment activation factor did not directly activate the SEEKING Command System. Oxytocin should mediate the release of relatively high amounts of 5-hydroxytryptamine^30^ in order to increase the level of dopamine (*vide supra*), thereby activating SEEKING. Since oxytocin signaling is predicted here to be evoked via action of sensory nerves for the onset of attachment (Figure 1, *vide supra*), the *caudal nucleus tractus solitaries* can be classified tentatively as the brain-specific area of an underlying attachment drive (Table 1). The fact that oxytocin can address in a direct manner other Command Systems cannot be taken as an argument against the view of attachment being a drive, because imperative motor factors of Freudian drives are also known to possess such ability (Kirsch, 2019).

In any case Bowlby’s proposition to classify attachment as an instinctive behavior runs into serious difficulties. The authors follow the view that an instinct has an innate nature and emerges without any training, learning or education processes (Maslow, 1954; Spink, 2010; Blumberg, 2017; Adolphs and Anderson, 2018). The occurrence of such an impulse in human beings has been discussed very controversially (e.g., Maslow, 1943, 1954; Birney and Teevan, 1961), but observations that human infants (and in part older individuals) respond fearfully to (pictures of) snakes (Headland and Greene, 2011; Hoehl et al., 2017; Denzer, 2018) represent at present the best indication of such operating instincts in humans. However, the situation is quite different in the social bonding of an infant to its mother (or, more generally-speaking to its care-giver), because an infant can differentiate other individuals by means of olfactory communication (e.g., Vaglio, 2009), and such an ability requires learning processes. Thus, the mother-infant tie is classified here as a drive,^31^ albeit not a Freudian one because this attachment drive operates consciously and is intensified (as opposed to deactivated) by 5-hydroxytryptamine (Figure 1, *vide supra*).

## Conclusion

Tinbergen’s assertion that his description of instinct is complementary to Freud’s theory of motivational drives indicated very strongly that Freud’s framework is also complementary to attachment urges (this is especially true for the mother-infant tie), because the concept of Hierarchical Organization of Circuit Nodes (Tinbergen, 1950) was used by Bowlby as a building block for his theory of instinctive behavior (*vide supra*). The complementary action of the mother-infant tie and Freudian drives is evident at the level of initiating factors (Table 1), at the level of Command Systems,^32^ and even at the level of signal transduction. After the concerted protest of A. Freud, M. Schur and R. Spitz (*vide supra*), Bowlby not only lost his interest in imposing the mother-infant tie as a compatible framework to Freud’s theory of motivational drives but had the need to create a superior explanation of the human mind (at all theoretical costs). In doing so, Bowlby deprecated the “drive” construct as a fundamentally descriptive placeholder of a sequence of behavior (*vide supra*) in contrast to his constructs of “instinctive behavior” with attachment as a prime example. The price to pay was the creation of doubts by ignoring both the reservation of many psychologists against the operation of (animal) instincts in human beings (e.g., Maslow, 1943, 1954) and also Tinbergen’s indication that animal instincts operate consciously whereas Freudian forces act in an unconscious manner. Based upon the views that both a drive activates the SEEKING command system and the mother-infant tie requires learning processes, Bowlby’s view is revisited here and the mother-infant tie is classified as a drive. Thus, four essential drives are at work in the neonate: thirst, hunger, sleep and attachment. Of course, a ranking order of importance of the drives, as had been introduced by both Bowlby and Anna Freud^33^, is of no benefit because all these drives are in the service of infant survival and should therefore assist each other. This is able to explain the occurrence of high concentrations of the imperative motor factors of thirst and hunger in the infant at birth and in the early postnatal period (*vide supra*) because they can both result in a release of oxytocin, i.e., the initiating factor of attachment activation in women and infants (Figure 1). Paradigmatically, the mother-infant tie is seen as a typical example of attachment. By considering the fact that the mother-infant tie is (for example on the level of intermediary oxytocin and vasopressin, respectively, and most likely also on the level of activated Command Systems) quite different from the affective relationship in pair bonding, it would be realistic to conclude that a variety of attachment drives are at work in human beings. Due to such uncertainties the proposed mechanism is tightly restricted to the mother-infant tie from the perspective of the infant (Figure 1). It should be noted that this mechanism supports entirely Harlow observations (i.e., social bonding is (temporary) more important than hunger, *vide supra*) because the initiating factor of the mother-infant tie (i.e., oxytocin) can down-regulate the claim of the hunger-drive by decreasing the efficacy of its corresponding imperative motor factor (i.e., ghrelin, *vide supra*).

Our evaluations are limited because we focused strictly on early interactions between the mother-infant tie and Freudian drives (*vide supra*). By doing so various late down-streaming intermediates (e.g., a variety of neurotransmitters and the majority of endorphins) with high importance for a drive activity but less importance for the interaction of the drives were in this manuscript less well incorporated. Due to this selection the hypothesis outlined in Figure 1 (*vide supra*) cannot be used to justify any pharmacological interventions, e.g., an intranasal application of oxytocin in order to intensify the mother-infant tie is not justified by the proposed mechanism^34^. Nevertheless, the situation is quite different when an infant receive maternal ghrelin via breast-feeding (*vide supra*). At first, this experimental finding seems to be a little bit bizarre because the puzzler arises why a hungry infant should receive the imperative motor factor of hunger from its mother? The neonatal gastrointestinal tract exhibits less proteolytic activity and is more permeable to proteins than in older individuals (Banks et al., 1983). Because of this reduced proteolytic activity, the maternal peptide ghrelin can reach the neonatal bloodstream by crossing the gastrointestinal tract during the early postnatal period. Since the drive-specific brain area of the mother-infant tie is activated during breast-feeding, maternal ghrelin should be able to intensify (in the infant) the mother-infant tie according to the following diagram (Fig. 1). Such an action is in agreement with Tronick’s theory that the mother “*is part of the infant’s homeostatic regulatory system”* (Tronick, 2007, p. 403 f.).

Of course, the experimental findings summarized in Figure 1 may represent only a superficial insight into a potential operation of the mother-infant tie but it has been clearly demonstrated that the action of Freudian drives cannot be marginalized for an extensive understanding of the composition of more complex drives like the ones responsible for attachment. As a result, the rigorous dispute between attachment theory and psychoanalysis should be revisited: “*Now – and not entirely through attrition – we seek to locate the debate not in terms of bad blood, but of the need for new blood. Both attachment and psychoanalysis must, if either or both fields are to retain their intellectual vigor and relevance, rethink their approach to psychopathology in a manner that moves us on from a descriptive, category-driven approach, the legacy of an essentially nineteenth-century medical mind-set.”* (Fonagy and Campbell, 2015, p. 245). The rethinking we outlined here proposes to follow the inclusion of a “mother” as part of the child’s regulatory system and extend this line of reasoning to other social beings in later life. This means, to re-include the social-interactive dimension in order to vault over conceptual individualism.

## Author Contributions

MK had the original idea, introduced biochemical/endocrino- logical knowledge, wrote 75% of the text and revised the entire manuscript. MB introduced important psychological/psychanalytical knowledge, especially in relation to Freudian/Bowlbyian perspective and wrote 25% of the text.

## Conflict of Interest

The authors declare that the research was conducted in the absence of any commercial or financial relationships that could be construed as a potential conflict of interest.

## Footnotes

1 Freud pointed out (S. Freud, 1905, p. 1537): “*Anxiety in children is originally nothing other than an expression of the fact that they are feeling the loss of the person they love*,.”

2 Bowlby noted (Bowlby, 1969, p. xviii): “*In 1951, at a sensitive phase in my thinking about problems of separation, Julian Huxley fanned a germinal interest in ethology and introduced me to the published classics of Konrad Lorenz and Niko Tinbergen. To all three I am grateful for continuing my education and for encouragement.*”

3 Stress exposure has been shown to increase endogenous opioid levels of the dynorphin family (Nabeshima et al., 1992) and dynorphins are involved during social stress induced by maternal separation (i.e., detachment) in experimental animals during early postnatal life (Michaelis and Holtzman, 2008).

4 Bowlby noted (Bowlby, 1969, p. 88): “*Before we examine further the role of environmental factors, it may be useful to consider briefly the organization of behavior by means of causal hierarchy, a principle to which* Tinbergen (1950) *first drew attention*.”

5 Obviously because “unconscious” refers in medicine to brain activities during senseless states, Tinbergen replaced the term “unconscious” with “non-conscious”.

6 Bowlby proposed the theory of instinctive behavior as a superior alternative to Freud’s theory of motivational drives (Bowlby, 1969, p. 220).

7 Bowlby noted (Bowlby, 1969, p. 179): “*It postulates that the child’s tie to his mother is a product of the activity of a number of behavioral systems that have proximity to mother as a predictable outcome*.”

8 The introduction of feedback stimuli was for Bowlby a significant advantage over Freud’s theory of motivational drives (e.g., “*As a result the concept of feedback has never been exploited in psychoanalytic theorizing,.*” (Bowlby, 1969, p. 23)).

9 In 1999 the possibility of hormone-controlled drives was covered by Pfaff (1999).

10 Noticeably, persistent mistranslations of Freud’s terms (pressure-dependent) *motorisches Moment* (correctly, in the Freudian sense: imperative motor factor) as “motor factor”, *Trieb* (correctly: drive) as “instinct”, *Trieblehre* (correctly in the Freudian sense: theory of motivational drives) as “theory of instincts” and *unbewusst* (correctly: subconscious) as “unconscious” have given rise to a variety of misunderstandings (Kirsch, 2019).

11 These hormones can be understood as the “springs” of the corresponding drives.

12 The classification of Command Systems was evaluated according to Watt (2017). The following major structures were given as constituents of the seven Command Systems. SEEKING: *ventral tegmental area, lateral hypothalamus, periaqueductal gray, nucleus accumbens*. RAGE: *medial amygdala, bed nucleus of stria terminalis, anterior and ventromedial and perifornical hypothalamus, dorsal periaqueductal gray*. FEAR: *central and lateral amygdala, medial hypothalamic to dorsal periaqueductal gray to nucleus reticularis pontine caudalis*. LUST: *bed nucleus of stria terminalis, corticomedial amygdala to preoptic and ventromedial hypothalamus to lateral ventral periaqueductal gray*. CARE: *anterior cingulate to bed nucleus of stria terminalis to preoptic hypothalamic to ventral tegmental area to more ventral periaqueductal gray*. PANIC: *anterior cingulate, anterior thalamus to bed nucleus of stria terminalis, ventral septum to midline and dorsomedial thalamus to dorsal preoptic hypothalamus to more dorsal periaqueductal gray*. PLAY: *parafascicular/centromedian thalamus, dorsomedial thalamus, posterior thalamus, projecting to ventral periaqueductal gray* (septum inhibitors).

13 The organ-dependent secretion of an imperative motor factor represents an internal stimulus.

14 The “spring” of the mother-infant tie stream.

15 Experiments with monogamous animals demonstrated that the peptide vasopressin is a main regulator of male behavior whereas oxytocin is more important for female behavior. Nevertheless, the view that vasopressin and oxytocin mediate exclusively behavior of males and females, respectively, is a severe oversimplification (Freeman and Young, 2013).

16 The *paraventrucular nucleus* of the *hypothalamus* sends oxytocinergic projections to the *nucleus tractus solitaries* (Torres Uchoa et al., 2013) whereas the *caudal nucleus tractus solitaries* projects catecholaminergic neurons to the *supraoptic nuclei* in order to evoke oxytocin secretion (Raby and Renaud, 1989). Noticeably, suckling evokes oxytocin release in the *supraoptic nucleus* of rats (Moos et al., 1989). Nevertheless, there are long –range projections of oxytocinergic neurons between the *supraoptic nucleus* and the *paraventricular ones* within the *hypothalamus*, suggesting potential communication between them (Mitre et al., 2018).

17 The application of oxytocin in virgin rats evoked the onset of maternal behavior and such a pharmacological manipulation failed by blocking the targets (i.e., so-called receptors) for oxytocin in the brain (Pedersen et al., 1982, 1994). Baby rats seek out their mothers by favoring areas of maternal odors and such a search can be largely repressed by blocking oxytocin receptors in the brain (Nelson and Panksepp, 1996).

18 The amount of oxytocin is very high in both human mothers and their infants one minute after birth (Uvnäs Moberg and Prime, 2013).

19 The self-stimulation effect (a seldom phenomenon of brainchemicals) of oxytocin, as mentioned by Theodosis (2002), was experimentally verified by Carson et al. (2010).

20 5-Hydroxytryptamine is exclusively called serotonin [= serum vasoconstrictor (Rapport et al., 1948)] when it circulates as a hormone in the periphery, whereas a centrally operating entity is also referred to as 5-hydroxytryptamine (Amin et al., 1954).

21 The amines of interest are noradrenaline, dopamine and 5-hydroxytryptamine (*vide supra*).

22 There are multiple evidences that estradiol increases the density of oxytocin receptors in the brain and enhances the affinity of oxytocin to its corresponding receptors (Jirikowski et al., 2018).

23 Oxytocin is metabolized by various oxytocinases present in synaptic brain membranes (Stancampiano et al., 1991). Because of this observation it can be assumed that oxytocinases were constitutively (i.e., permanently) active. Therefore, any change in the rate of oxytocin release would sensitively change the concentration of oxytocin and any inhibitor of oxytocin release can therefore be classified as an attachment termination factor.

24 This interaction (ghrelin leads to oxytocin release that can lead to the decrease of ghrelin) may represent a feedback mechanism for hunger with oxytocin as a feedback stimulus (Figure 1 *vide infra*).

25 Hypothalamic oxytocinergic neurons are generated in humans in the middle of pregnancy (Swaab, 1995).

26 In mammal pups, oxytocin is released during the first sucking period, which facilitate the preference for the mother (Nowak et al., 2011). Accordingly, tactile contacts between newborn animals and their mothers activates the signaling of oxytocin (Muscatelli et al., 2018).

27 As noted in Table 1, oxytocin did not always down-regulated the *central amygdala* and thereby the command systems FEAR in human women because there are distinct situations (overviewed in Wigton et al., 2015) where oxytocin operates in an opposite manner.

28 The amounts of β-endorphin and of its precursor are very high in both human mothers and their infants during the first 24 h of neonatal life (Facchinetti et al., 1982).

29 Wright and Panksepp noted: “*The ability to process and* ‘*decide*’ *between the drives might be lost if each drive is not also an independent generator. In other words, we have to sustain drive-specificity.*” (Wright and Panksepp, 2012, p. 18)

30 The view that oxytocin always acts via intermediary 5-hydroxytryptamine should be discarded. For example, oxytocin induces penile erection in male rats by increasing glutamatergic neurotransmission (Succu et al., 2011; Love, 2014).

31 Bowlby pointed out that his behavioral systems are goal-corrected and this ability required “*receiving and storing instructions regarding the set goal*”(Bowlby, 1969, p. 70). Thus, the concept of “instinctive behavior” dependents on learning processes and Bowlby most likely refrained from the classification “drive” in order to avoid any Freudian nomenclature.

32 The initiating factor of the mother-infant tie and the ones of the Freudian drives are opponents of the *central amygdala* (FEAR) activity. While oxytocin inhibits the *central amygdala* (Huber et al., 2005; Viviani et al., 2011), imperative motor factors (i.e., adenosine, angiotensin II, ghrelin and testosterone) activate this brain area (Goodman and Snyder, 1982; Simerly et al., 1990; Lenkei et al., 1996; Cruz et al., 2013; Yoshimoto et al., 2017).

33 “*He* [Bowlby] *sets up a controversy between the tie to the mother and the action of the pleasure principle in terms of “primary and secondary drive” and criticizes us for reversing their order of importance, i.e., for regarding the tie to the mother as a secondary, the search for pleasure as a primary instinctual urge.*” (A. Freud, 1960)

34 At present, it is unclear whether exogenously oxytocin has abuse potential because endogenously oxytocin leads to β-endorphin secretion (Kovatsi and Nikolaou, 2019). Synthetic oxytocin agonists and exogenous oxytocin in a high dose evoked euphoria in experimental animals (Ring et al., 2010; Laszlo et al., 2016).
